# Cofactor recycling strategies for secondary metabolite production in cell-free protein expression systems

**DOI:** 10.1007/s12551-024-01234-1

**Published:** 2024-09-26

**Authors:** Yutong Zou, Constance B. Bailey

**Affiliations:** School of Chemistry, University of Sydney, Sydney, NSW Australia

**Keywords:** Cell Free Protein Synthesis, Cofactor recycling strategies, Secondary metabolite production

## Abstract

Cell-free protein synthesis (CFPS) has emerged as an attractive platform for biotechnology and synthetic biology due to its numerous advantages to cell-based technologies for specific applications. CFPS can be faster, less sensitive to metabolite toxicity, and amenable to systems that are not easily genetically manipulated. Due to these advantages, a promising application of CFPS is to characterize biosynthetic gene clusters, particularly those harbored within the genomes of microorganisms that generate secondary metabolites, otherwise known as natural products. In the postgenomic era, genome sequencing has revealed an incredible wealth of metabolic diversity. However, far more of these pathways are termed “cryptic,” i.e., unable to be produced under standard laboratory conditions than have been characterized. A major barrier to characterizing these cryptic natural products using CFPS is that many of these pathways require utilization of complex cofactors, many of which to date are not recycled efficiently or in an economically viable fashion. In this perspective, we outline strategies to regenerate cofactors relevant to secondary metabolite production in CFPS. This includes adenosine 5′-triphosphate, coenzyme A, redox cofactors (iron-sulfur clusters, nicotinamide adenine dinucleotide phosphate, flavin adenine dinucleotide), all of which play a crucial role in important biosynthetic enzymes. Such advances in cofactor recycling enable continuous production of complex metabolites in CFPS and expand the utility of this emergent platform.

## Introduction

Cell free protein synthesis (CFPS) systems have long had a role in elucidating fundamental science in biochemistry and molecular biology. The earliest iteration of cell free synthesis was reported in 1907 by Nobel laureate Edaurd Buchner (Nobel Prize in Chemistry 1907) who demonstrated bio-ethanol production in yeast cell extracts, pioneering contributing to the development of CFPS (Buchner 1897). Another example of a Nobel Prize was the groundbreaking work of Nirenberg and colleagues deducing of genetic code in *Escherichia coli* cell extract (Nirenberg and Matthaei 1961). In more recent years, CFPS has been applied to prototype expression and undergo biomanufacturing applications (Hodgman and Jewett 2012). Without the cellular survival objectives, CFPS significantly shortens the protein expression process and can be applied towards optimizing specific targets of expression (Carlson et al. 2012; Sword et al. 2024). By supplementing cell extracts with amino acids, nucleotides, and secondary energy substrates, protein synthesis can be initiated in vitro using the native cellular transcriptional and translational machinery. In the absence of a cell membrane, the process of protein synthesis can be directly monitored, rapidly sampled, and directly modified (Silverman et al. 2020). Despite these advances, several obstacles have prevented their engineering potential in protein production, including low production yields, high energy expense, and no viable scale-up methods existing (Swartz 2006). However, the rapid technical development of synthetic biology in the last decade provides new possibilities for cell free biocatalysis and has addressed these limitations. Swartz and colleagues have reported a novel method for cell free system scale up and reached 100 L (Voloshin and Swartz 2005). Similarly, Sutro Biopharma, Inc. demonstrated high productivity of cost-effective cell free protein synthesis at the hundred liters reaction (Zawada et al. 2011). Recent advances demonstrated that a eukaryotic CFPS platform has been developed based on *Nicotiana tabacum* BY-2 cell culture (BY-2 lysate; BYL), which scales the reaction volume across a 20,000 × range and is applied in a liter-scale reaction in a commercial bioreactor (Gupta et al. 2023). These milestones show that the CFPS system has the potential to become an industrially scaled platform for producing recombinant DNA proteins.

**Fig. 1:**
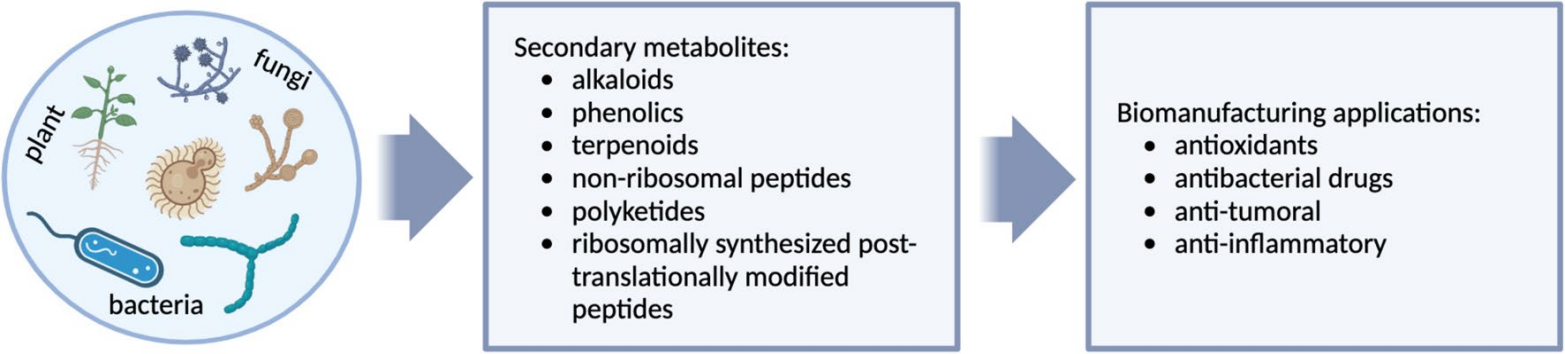
Schematic diagram of secondary metabolites sources, classes, and biomanufacturing applications

While applications of protein expression have expanded in cell free systems, one area that is an emergent application developed in recent years has been towards metabolite production. Numerous secondary metabolites gene clusters have been discovered in plant and microbial genomes and have important pharmacological applications (Fig. 1). In particular, the postgenomic era has revealed a vast wealth of untapped metabolic diversity harbored within microorganisms, most of which is called “cryptic” or “silent” inasmuch as it is not produced under standard laboratory culture conditions and thus are unable to be isolated or characterized (Ji et al. 2024; Zhao et al. 2024). A challenge in the characterization of silent is that in their natural context, secondary metabolites are produced for precise ecological reasons that are not always able to be easily recapitulated in a laboratory environment. Many metabolites are toxic when produced above physiological concentrations. For all these reasons, many metabolites that may have promising bioactivity or interesting structural diversity remain uncharacterized. A separate but related application is that the biosynthetic enzymes to generate secondary metabolites frequently harbor unusual and remarkable biocatalytic transformations, including rare domains or domains of unknown function (DUFs) which perform chemistry inaccessible via chemical synthesis (Li et al. 2024).

A CFPS system contains crude cell lysates/extracts and DNA supplemented with associated energy sources, nucleotides, amino acids, salts, and cofactors, which are essential for the reaction to generate the desired product. It is important to emphasize that there is a difference with one-pot cell free production which uses multiple purified enzymes, substrates, and cofactors but no DNA input. Most of the enzymes in current CFPS system use are cofactor-dependent such as oxidoreductases and transferases which can perform complex chemistry and catalyze many synthetically useful reactions (Zhao and van der Donk 2003). The biosynthesis and regeneration of cofactors are expensive for the cell and are typically required in stoichiometric amounts for enzymatic transformations (Bergquist et al. 2020). Therefore, cofactors must be regenerated and recycled to improve the CFPS efficiency through a redesign of metabolic pathways, considering the economic feasibility of CFPS (Ullah et al. 2023). In this review, we discuss cofactor recycling strategies particularly relevant to the generation of secondary metabolites. This includes ATP, coenzyme A, and redox cofactors (iron sulfur clusters, NADPH, and flavins). Taken together, cofactor regeneration considerations are necessary to advance the characterization of secondary metabolites with CFPS.

## Adenosine 5’-triphosphate (ATP) regeneration

ATP-related functions include the synthesis of dNTP, aminoacyl-tRNA charging, and regeneration of GTP in the peptide bonding process (Jewett and Swartz 2004). In the biosynthesis of many classes of natural products, ATP is used as a phosphorylating agent. YcaO enzymes are widely studied ones to catalyze phosphorylation of peptide backbone amide bonds in the biosynthesis of ribosomally synthesized and post-translationally modified peptides (RiPPs) (Franz et al. 2017). YcaO-catalyzed reactions have been found in the formation of azolines (Burkhart et al. 2017), amidines (Travin et al. 2018), and macrolactamidines (Franz et al. 2017). In the biosynthesis of nonribosomal peptides, adenylation domains catalyze the selective incorporation of acyl building blocks. Through activating a specific acyl substrate with ATP, the domain forms an acyl adenylate intermediate and then transfers the acyl group onto the carrier protein domain (Miyanaga et al. 2022). Therefore, the ATP availability will obviously limit the enzyme-catalyzed biotransformation. Whitesides first reported a series of useful ATP-recycling reactions and methods in the 1970s and 1980s (Crans and Whitesides 1983; Pollak et al. 1977). Three enzymatic methods are the most popular: phosphoenolpyruvate (PEP) catalyzed by pyruvate kinase; acetylphosphate coupled with acetate kinase; and polyphosphate coupled with polyphosphate kinase (PPK) (Zhao and van der Donk 2003).

### Acetate kinase/acetyl phosphate

**Fig. 2:**
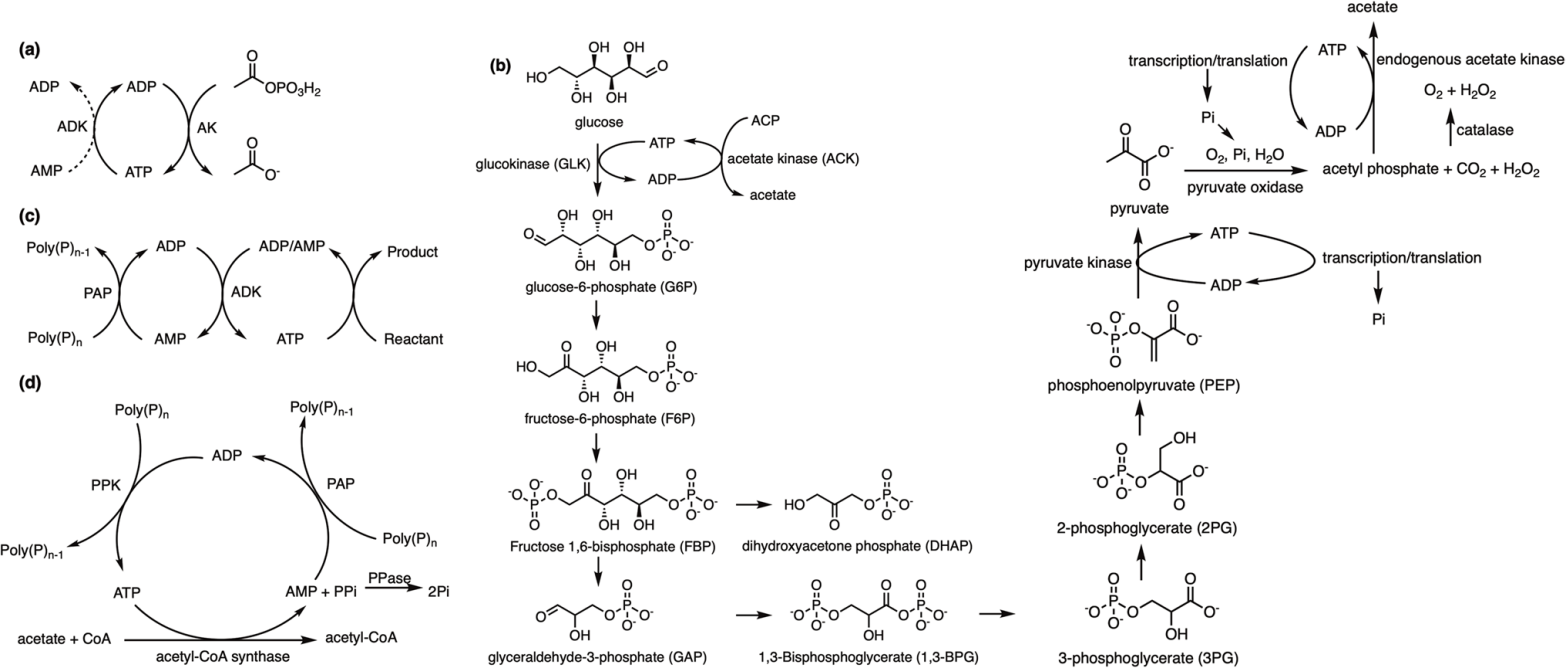
Mechanisms of different ATP regeneration systems. **a**, ATP regeneration using acetyl phosphate and acetate kinase (adapted from Bartzoka et al. 2022); **b**, glycolysis pathway and ATP regeneration using glucose-6-phosphate, phosphoenolpyruvate, and pyruvate as the energy sources (adapted from Kim and Kim 2009; Yan et al. 2014); **c**, ATP regeneration from AMP and poly(P) by the PAP-ADK system (adapted from Resnick and Zehnder 2000); **d**, ATP regeneration from AMP by the PAP-PPK system combined with the acetyl-CoA synthesis. ADK, adenylate kinase (adapted from Zhao and van der Donk 2003); AK, acetate kinase; PAP, polyphosphate AMP phosphotransferase; PPK, polyphosphate kinase; PPase, inorganic pyrophosphatase

ATP is synthesized and recycled from diphosphate (ADP) and/or monophosphate (AMP) through an auxiliary enzymatic reaction (acetate kinase) using acetyl phosphate as the phosphate donor (Fig. 2a). Lee and colleagues evaluated ATP regeneration with acetyl phosphate and investigated the production of sugar nucleotides in *E. coli*. Their initial attempts using endogenous acetate kinase-catalyzed ATP recycling showed a positive result, which is also economically attractive since acetate kinase is the most abundant enzyme in *E. coli*, and acetyl phosphate is a cheaper substrate (Lee et al. 2002). The subsequent findings reported that overexpression of the acetate kinase activity improved the yield (Lee et al. 2010). In another study, Easton and coworkers developed rapid regeneration of ATP from AMP with acetyl kinase and applied this to both polymerase chain reaction (PCR) to produce DNA and cell-free protein synthesis of mRNA (Alissandratos et al. 2016). They reported the conversion from dNMP to dNTP in the presence of acetyl phosphate, ATP recycling, and endogenous kinases. This work provides an alternative strategy in economics and is environmentally friendly since it overcomes the low dNTP yield and the usage of toxic chemical solvents in current chemical production (Bao and Ryu 2007).

### Pyruvate kinase/phosphoenol pyruvate and glycolytic intermediates

One of the most widely used ATP recycling systems is the PANOx system, first developed by Swartz. The PANOx system uses phosphoenolpyruvate (PEP) to drive the regeneration of ATP (Fig. 2b) (Swartz 2006). Drawbacks to this recycling method include the short reaction duration (~ 60 min) of ATP bursts, which causes the accumulation of inhibitory phosphates which is subsequently inhibitory to cell-free protein synthesis (Kim and Swartz 2001). To address these limitations, new ATP regeneration approaches focus on glycolytic intermediates such as pyruvate and glucose-6-phosphate (G6P) rather than PEP as energy sources (Fig. 2b). G6P is the first glycolysis intermediate and can be effectively used for ATP regeneration (Ullah et al. 2023). Kim and Swartz found that G6P had a much lower cost and a higher potential for ATP generation than PEP (Kim and Swartz 2001). They reported a high yield result of protein synthesis by using G6P as a secondary energy source compared to PEP by optimizing the pH of the reaction mixture. G6P can prolong the reaction period, which results in more ATP being readily available. The successful usage of G6P in cell free reactions indicates that glucose is also available to regenerate ATP (Calhoun and Swartz 2005). Pyruvate is the end-product of glycolysis and several studies have demonstrated that it can be used for ATP regeneration (Fig. 2b) (Kim and Swartz 1999, 2001; Ullah et al. 2015). Kim and colleagues introduced pyruvate oxidase into the reaction which catalyzed the condensation of pyruvate and inorganic phosphate and produced acetyl phosphate. Endogenous acetate kinase then converted the acetyl phosphate to acetate and catalyzed the ATP regeneration. This pyruvate oxidase-dependent system was able to extend the duration of protein synthesis and prevent inorganic phosphate accumulation as well (Kim and Swartz 1999). Additionally, the usage of pyruvate significantly reduces the energy cost compared to the conventional energy source (PEP). However, there are two limitations of this method to be easily scaled up. Pyruvate oxidase is an exogenous enzyme from *Lactobacillus* or *Pediococcus sp.* and is not present in *E. coli* cell extracts. Synthetic biology can further optimize by remove the necessity to add exogenous pyruvate oxidase by cloning the encoding gene into a plasmid vector and heterologously expressing it in *E. coli*. Another limitation is the requirement of oxygen utilized by pyruvate oxidase for the conversion from pyruvate into acetyl phosphate (Kim and Swartz 2001; Kim and Kim 2009). Kim and Swartz bypassed these limitations by adding cofactors, NAD, and CoA. In this process, pyruvate dehydrogenase converts pyruvate to acetyl-CoA after the addition of NAD and CoA. Acetyl-CoA is subsequently converted to acetylphosphate, thus ATP regeneration is stimulated when acetylphosphate produces acetate (Kim and Swartz 2001). The utilization of the first intermediate (G6P) and the downstream products (PEP and pyruvate) in the glycolysis pathway also inspired the examination of other intermediates. There have been several studies that reported the successful results of the glycolytic intermediates used as efficient energy sources such as fructose-1,6-bisphosphate and 3-phosphoglycerate (Kim et al. 2007; Sitaraman et al. 2004).

### Polyphosphate kinase/polyphosphate

The use of inorganic polyphosphate (poly(P)) to regenerate ATP has attracted significant attention because of its low cost, high stability, and serve as an attractive phosphoryl donor (Iwamoto et al. 2007). An *E. coli* recombinant producing *Thermus* polyphosphate kinase (PPK) has been reported to regenerate ATP when treated with exogenous polyphosphate and ADP (Fig. 2c). The *E. coli* was also heat-treated to allow the membrane penetration of ATP and poly(P) into the cell. This system was applied to generate fructose 1,6-diphosphate (FDP) and further tested and optimized by employing the production of glycerol 3-phosphate (G3P) from glycerol (Iwamoto et al. 2007; Restiawaty et al. 2011). In another study, Resnick and Zehnder designed an ATP regeneration system using poly(P), polyphosphate AMP phosphotransferase (PAP), and polyphosphate-independent adenylate kinase (ADK). PAP and ADK are from *Acinetobacter johnsonii* 210A which harbors GC content and thus poses difficultly cloning, which could be mitigated by codon replaced via gene synthesis. In this method, polyphosphate AMP phosphotransferase catalyzes the conversion from AMP to ADP and ADK regenerates ATP and AMP from ADP molecules. The system was also used to synthesize glucose-6-phosphate with hexokinase (Resnick and Zehnder 2000). Similarly, Kameda and colleagues developed a method to regenerate ATP from AMP and poly(P) catalyzed by PAP and a recombinant PPK (Fig. 2d). This method also combined with acetyl-CoA synthesis using acetyl-CoA synthase and supplemented with inorganic pyrophosphatase (PPase) which minimized the inhibitory effect of PPi and drive the regeneration system. This strategy provides a higher total turnover number for ATP and has the advantage of regenerating GTP from GMP compared with the PAP-ADK system of Resnick and Zehnder (Kameda et al. 2001; Resnick and Zehnder 2000).

### Future prospects for ATP recycling: histidine kinase

Recently, a novel mechanism for ATP synthesis was uncovered by Jiang and coworkers from a bacterial histidine kinase domain HK853. The soluble domain of HK853 was shown to work effectively in vitro to generate ATP under mild conditions via recycling of ADP utilizing a substrate such as phosphorylated amino acids or phosphorylated glycerol. While it has not been explored in cell free systems, this could be a valuable tool to add to the toolkit for improved ATP recycling such that it is not such a limitation in cell free systems (Ji et al. 2024).

## Coenzyme A (CoA)

Coenzyme A (CoA) is a ubiquitous essential cofactor in all domains of life and serves as an acyl group carrier and carbonyl activating group. It plays an important role in a variety of metabolic transformations, including the biosynthesis and catabolism of fatty acid, tricarboxylic acid cycle, and the biosynthesis of polyketides and nonribosomal peptides (Begley et al. 2001; Leonardi et al. 2005). As a mandatory cofactor, CoA is utilized by approximately 4% of enzymes, and it is involved in more than 100 different intermediate metabolic reactions (Jackowski 1996; Lee and Chen 1982). It is of particular importance as it serves as an important key posttranslational modification for the phosphopanetheinylation necessary for polyketide synthases (PKSs) and nonribosomal peptide synthetases (NRPSs) as well as the primary building blocks (e.g. acetyl-CoA, propionyl-CoA, malonyl-CoA, methylmalonyl-CoA) for polyketide synthases (Crosby and Crump 2012). CoA-dependent anabolic routes normally have three steps, including activating low energetic substrates and yielding an acyl-CoA derivative, modifying the acyl-CoA derivative through enzymatic steps, and releasing the target product by removing CoA (Ducrot et al. 2024).

### CoA recycling in the butanol pathway

**Fig. 3:**
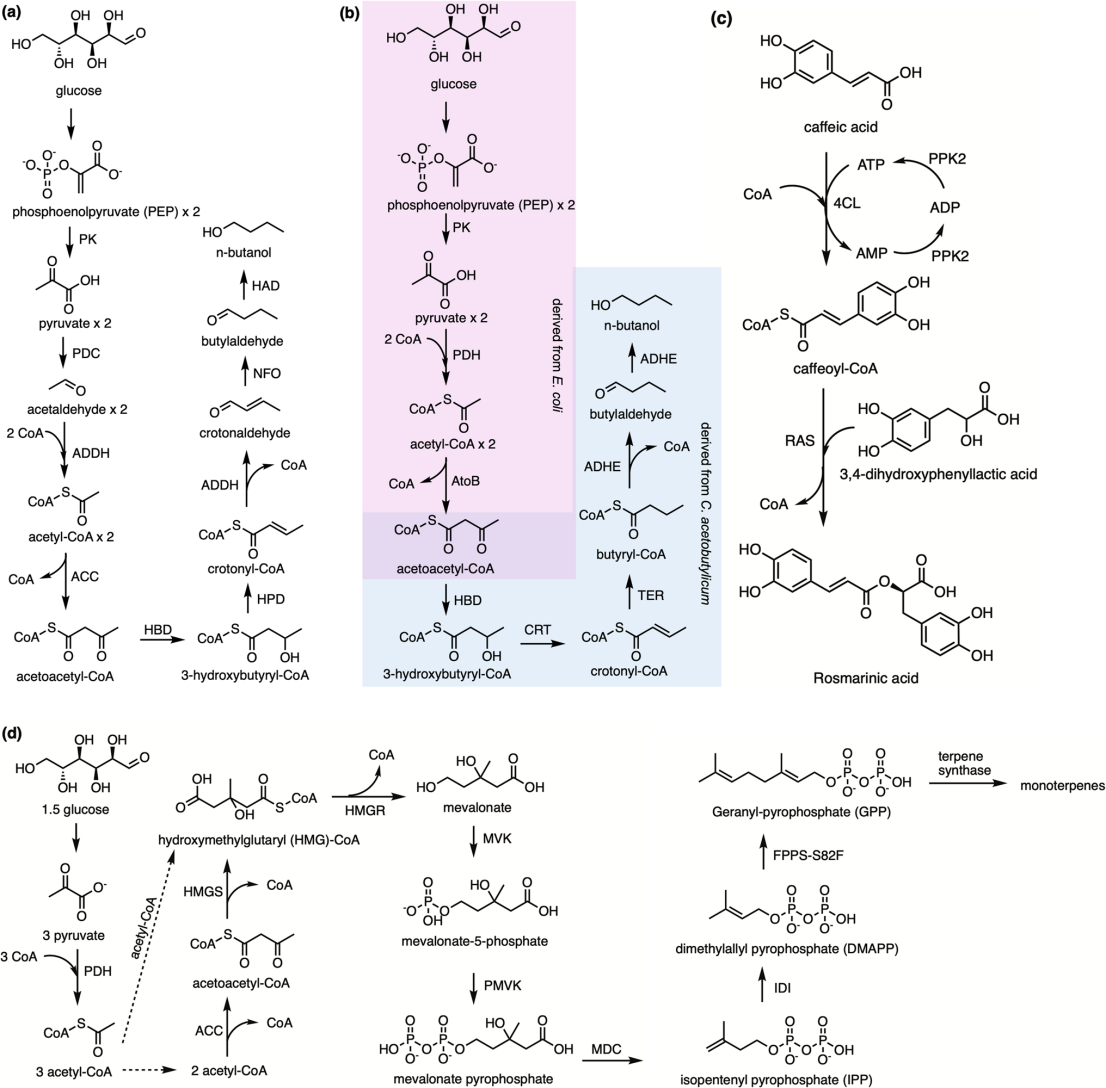
Schematic of CoA recycling in different pathways. **a**, the cell free pathway of n-butanol from glucose (adapted from Krutsakorn et al. 2013); **b**, the constructed biosynthetic pathway of n-butanol. Acetyl-CoA is produced from glycolysis pathway in *E. coli*, then utilized by CoA-dependent pathway in *C. acetobutylicum* to produce n-butanol (adapted from Karim and Jewett 2016); **c**, rosmarinic acid biosynthesis pathway with CoA and ATP regeneration (adapted from Yan et al. 2019); **d**, biosynthetic pathway for conversion of glucose to monoterpenes. PK, pyruvate kinase; PDC, pyruvate decarboxylase (adapted from Korman et al. 2017); ADDH, CoA-acylating aldehyde dehydrogenase; ACC, acetyl-CoA acetyltransferase; HBD, hydroxybutyryl-CoA dehydrogenase; HPD, 3-hydroxypropionyl-CoA dehydratase; NFO, NADH-dependent flavinoxidoreductase; HAD, 3-hydroxyacyl-CoA dehydrogenase; PDH, pyruvate dehydrogenase; AtoB, thiolase; CRT, crotonase; TER, butyryl-CoA dehydrogenase; ADHE, bifunctional acetaldehyde/alcohol dehydrogenase; 4CL, 4-coumarate: coenzyme A ligase; RAS, rosmarinic acid synthase; PPK, polyphosphate kinase; HMGS, hydroxymethylglutaryl-CoA synthase; HMGR, hydroxymethylglutaryl-CoA reductase; MVK, mevalonate-5-kinase; PMVK, phosphomevalonate kinase; MDC, mevalonate pyrophosphate decarboxylase; IDI, isopentenyl pyrophosphate isomerase; FPPS-S82F, farnesyl-pyrophosphate synthase-S82F mutant

As a key example of the utility of CoA recycling, Krutsakorn and co-workers constructed the butanol pathway using 16 enzymes (Fig. 3a). The pathway first produced acetaldehyde from glucose through glycolysis and pyruvate decarboxylation, followed by CoA-acylating aldehyde dehydrogenase converting acetaldehyde into acetyl-CoA. Acetyl-CoA acts as the first intermediate for butanol synthesis, then two molecules of acetyl-CoA produce acetoacetyl-CoA by condensation. Subsequent reduction and dehydration were catalyzed by specific enzymes, which converted acetoacetyl-CoA into crotonyl-CoA and CoA was finally removed by CoA-acylating aldehyde dehydrogenase to yield crotonaldehyde, which is further reduced to the terminal alcohol (Krutsakorn et al. 2013). In another case, Karim and Jewett constructed a similar butanol biosynthetic pathway involving CoA intermediates and achieved rapid prototyping by accelerating design-build-test cycles (Fig. 3b). However, they produced precursor acetyl-CoA by using endogenous glycolytic enzymes and pyruvate dehydrogenase in the *E. coli* cell extracts; thus, the activation module is catalyzed by the pyruvate carboxylase rather than CoA-acylating aldehyde dehydrogenase reported by Krutsakorn et al. Crotonyl-CoA is also further reduced to butyryl-CoA, and CoA-deacetylating aldehyde reductase is subsequently removed CoA to generate butyraldehyde that is finally reduced by an alcohol dehydrogenase (Karim and Jewett 2016). These two pathways showed similar total turnover number of CoA (TTN_CoA_) with a value of 38 for Karim and Jewett and 35 for Krutsakorn, but almost six-fold titer of Karim and Jewett (20 mM) than 3.5 mM of Krutsakorn (Karim and Jewett 2016; Krutsakorn et al. 2013).

### CoA recycling in the production of rosmarinic acid

Rosmarinic acid is a hydroxycinnamic acid ester that is within an important class of phenolic natural products and has promising anti-carcinogenic and anti-tumorigenic properties (Bloch and Schmidt-Dannert 2014). The biosynthetic pathway of rosmarinic acid was established and engineered in *E. coli*, however, the yield was very low (Bloch and Schmidt-Dannert 2014; Jiang et al. 2016; Petersen et al. 2009). Yan and colleagues designed a CoA and ATP regeneration system for rosmarinic acid production through a four-enzyme cascade with high efficiency (Fig. 3c). The activation module is driven by 4-coumarate: coenzyme A ligase which catalyzed the conversion from caffeic acid to caffeoyl-CoA. 3,4-Dihydroxyphenyllactic acid was then introduced to condense with caffeoyl-CoA by rosmarinic acid synthase, producing rosmarinic acid and releasing CoA for recycling. ATP regeneration coupled with the formation of caffeoyl-CoA by adding polyphosphate kinases. The reaction conditions were also optimized to achieve a significantly high TTN for CoA and ATP, which were 444.5 and 820.6, respectively (Yan et al. 2019).

### CoA recycling in terpene biosynthesis

Korman and colleagues designed a platform to produce monoterpenes from glucose using up to 27 enzymes (Fig. 3d). These enzymes were purified individually and mixed in this system, supplementing substrate and cofactors for cell free production. Glucose was converted into pyruvate through the glycolysis pathway, followed by pyruvate dehydrogenase mediating the pyruvate activation and producing acetyl-CoA. The acetyl-CoA was modified through the mevalonate pathway to yield the target monoterpenes. The pathway started from the condensation of two molecules of acetyl-CoA and produced acetoacetyl-CoA. HMG-CoA synthases then catalyzed the generation of 3-hydroxy-3-methyl-glutaryl-coenzyme A (HMG-CoA). The CoA was removed by HMG-CoA reductase and produced mevalonate. Following the phosphorylation and rearranging of mevalonate, the generated isoprenoid precursors are ultimately converted to terpenes by condensation and cyclization. The platform produces various target monoterpenes by using the corresponding terpene synthase enzyme. The yield of monoterpene was in the range of 88–117 mM and TTN_CoA_ reached 357 (Korman et al. 2017).

## Redox cofactors

Oxidoreductases are widespread groups of biocatalysts, providing high activity and exquisite stereoselectivity in the synthetic strategy of pharmaceuticals, agrochemicals and food ingredients (Zheng and Xu 2011). They require redox cofactors such as NADPH, flavin, and iron-sulfur clusters to transfer energy in the form of reducing equivalents and keep redox homeostasis; thus, the regeneration of redox cofactors in CFPS is crucial (Chen et al. 2014). These cofactors’ recycling has already been further applied in the biotechnological production of valuable chemicals and synthetic biology (Shi et al. 2018; Wang et al. 2017; Weckbecker et al. 2010). A variety of strategies have been designed to optimize the yield of metabolites as well as support dynamic homeostasis between different redox states of cofactors (Partipilo et al. 2021).

### Iron-sulfur clusters

**Fig. 4:**
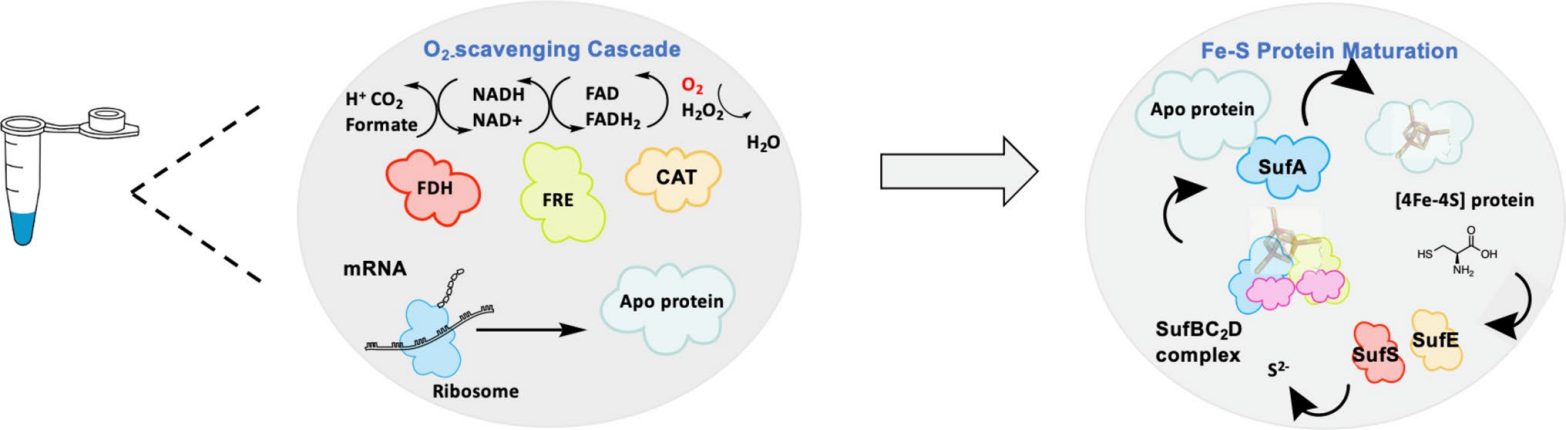
Schematic diagram of cell free mature [4Fe-4S] protein synthesis system. In vitro translation of mRNA was conducted with formate dehydrogenase (FDH), flavin reductase (FRE), and catalase (CAT). SUF pathway incorporates with [4Fe-4S] cluster to form the matured protein as the second step. Figure was adapted from (Wang et al. 2023)

Iron-sulfur clusters (Fe-S) serve as important prosthetic groups in many redox-active proteins and have been found in all types of organisms (Evans et al. 1966). One example that requires iron-sulfur cluster is radical S-adenosyl-l-methionine (SAM) enzymes, in which the iron-sulfur cluster coordinates their three cysteine residues, showing oxygen sensitivity in CFPS (Benjdia et al. 2017a, b). The radical SAM enzymes mediate key transformations in a variety of RiPPs pathways including thiopeptides (Kelly et al. 2009), epipeptides (Benjdia et al. 2017a, b), and bottromycins (Huo et al. 2012). Iron-sulfur clusters play a role in transporting electrons and are involved in numerous enzymatic reactions in metabolic and respiratory networks, radical chain reactions, DNA synthesis, protein translation, RNA modifications, and regulatory sensing (Lill and Freibert 2020). There are multiple Fe-S stoichiometries and coordination in which [2Fe-2S] and [4Fe-4S] types are the most common examples, and the [3Fe-4S] type is rarer (Beinert et al. 1997). Three helper protein systems have been developed for Fe/S protein assembly. The NIF (nitrogen fixation) system involved in Fe-S cluster assembly required nitrogenase to assimilate N_2_ (Jacobson et al. 1989). The ISC (iron-sulfur cluster) and SUF (mobilization of sulfur) systems are the more general ones for a variety of Fe-S protein assembly (Braymer et al. 2021; Zheng et al. 1998), while the SUF system is more O_2_-tolerant and resistant to iron starvation and oxidative stress (Boyd et al. 2014; Dai and Outten 2012). Swartz group first reported high-yield production of mature [2Fe-2S] Fd in a cell-free system. They utilized ISC helper proteins for production, but mature [2Fe-2S] Fd assembly was not facilitated by proteins probably because the ISC expression and activity were limited under aerobic conditions (Boyer et al. 2006). McGlynn and Fujishima developed a cell free mature [4Fe-4S] protein synthesis system using the SUF helper protein system and overcame the O_2_ lability of [4Fe-4S] by introducing an O_2_-scavenging cascade (Fig. 4). The SUF system can be dissected into three distinct steps, (i) S^2−^ extraction from cysteine by SufS-SufE pair, (ii), the assembly of the [4Fe-4S] cluster on SufBC_2_D complex, (iii), SufA transfer [4Fe-4S] cluster to recipient proteins. The SUF subunits in the SUF system and enzymes in the O_2_-scavenging system were individually purified, then reconstituted the SUF system stepwise in vitro and test the O_2_-scavenging system function in the PUREfrex system. These systems were added into the PUREfrex system as a one-pot, two-step reaction which was examined by using enzyme aconitase and thermophilic ferredoxin and yielded holo-aconitase of ∼0.15 mg/mL (Wang et al. 2023).

### Nicotinamide adenine dinucleotide phosphate (NADP(H))

**Fig. 5:**
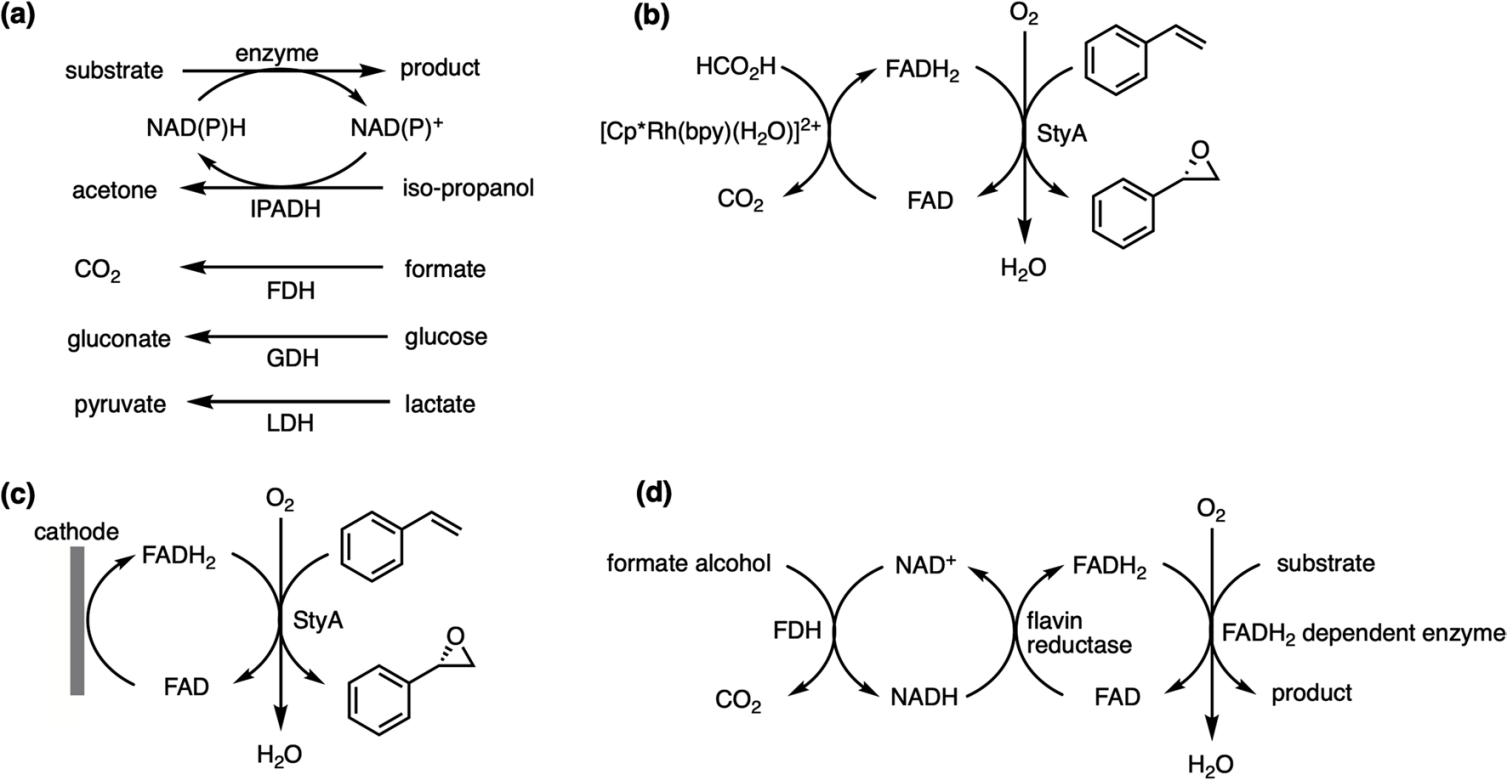
**a**, typical substrate-coupled approaches to regenerate NAD(P)H, ADH, alcohol dehydrogenase; FDH, formate dehydrogenase; GDH, glucose dehydrogenase; LDH, lactate dehydrogenase (adapted from Jia et al. 2022; Shi et al. 2018); **b**, chemoenzymatic approach with [Cp*Rh(bpy)(H_2_O)]^2+^ to regenerate FADH_2_ (adapted from Hollmann et al. 2003); **c**, electrochemical approach with artificial electrode to regenerate FADH_2_ (adapted from Hollmann et al. 2005); **d**, enzymatic approach using formate dehydrogenase (FDH) and flavin reductase to regenerate FADH_2_ (adapted from (Hofstetter et al. 2004)

NADP^+^ and NADPH are generally distributed redox cofactors, playing a major role in anabolism (Fukuzumi et al. 2018; Kim and Gadd 2008) and are necessary for many reductive steps in the biosynthesis of secondary metabolites such as polyketides and terpenes (Korman et al. 2017; Metsa-Ketela et al. 2013; Ng et al. 2015; Piasecki et al. 2011). It is important to note that cell-free systems must have NADP^+^/NADPH balance as part of their pathway design at the start of the cycle (Taniguchi et al. 2017; Zhang 2015). Therefore, an appropriate redox cofactor regeneration system is normally coupled with the reactions for chemical manufacturing to avoid stoichiometric consumption of cofactor and keep continuous utilization of substrate (Shi et al. 2018; Suryatin Alim et al. 2023). To date, the NAD(P)H regeneration systems include chemical, electrochemical, photochemical, and enzymatic methods (Immanuel et al. 2020; Shi et al. 2006; Wu et al. 2013). Enzymatic methods are most widely used due to their efficiency, reliability, and reproducibility and provide a high TTN value (Ullah et al. 2023). The most frequently used classification of enzymatic approaches uses another substrate and its respective enzyme. Common tool enzymes include alcohol dehydrogenase (ADH) (Jia et al. 2022), formate dehydrogenase (FDH) (Bozic et al. 2010), glucose dehydrogenase (GDH) (Xu et al. 2007), and lactate dehydrogenase (LDH) (Lee and Whitesides 1985) (Fig. 5a). These four representative enzymes drive the reaction to generate acetone, carbon dioxide, gluconate and pyruvate coupled with NAD(P)H regeneration. Additionally, NAD(P)H can be regenerated through cascade enzymes. An example is the 12-enzyme system developed by Wang and co-workers to produce NAD(P)H at a high yield of 11.4 mol NADPH per cellobiose (Wang et al. 2011).

### Flavin adenine dinucleotide (FAD)

Flavin adenine dinucleotide and dihydroflavine adenine dinucleotide (FAD/FADH_2_) serve as electron shuttles and are involved in many biocatalytic reactions including D-Amino acid oxidase (DAAO) catalyzed the oxidative deamination of D-amino acids (Pollegioni et al. 2008), succinate dehydrogenase (SDH) catalyzed succinate oxidation (Kim and Winge 2013), and glucose dehydrogenase (GDH) catalyzed glucose oxidation (Tsujimura 2019). Hollmann’s group first reported a direct regeneration method of flavin-dependent monooxygenases using the artificial organometallic complex [Cp*Rh(bpy)(H_2_O)]^2+^ (Fig. 5b). The FADH_2_-dependent monooxygenase StyA can be regenerated directly through redox catalysts [Cp*Rh(bpy)(H_2_O)]^2+^ which converts FAD into FADH_2_. By utilizing this cell-free chemoenzymatic approach, enantiopure epoxides can be synthesized asymmetrically (Hollmann et al. 2003). Complex [Cp*Rh(bpy)(H_2_O)]^2+^ has also been applied in FADH_2_-dependent halogenation (Unversucht et al. 2005). An electrochemical FAD/FADH_2_ regeneration system has been developed to investigate the biocatalytic asymmetric epoxidation by means of an artificial electrode (Fig. 5c). Other examples include the production of fumarate and xylose (Jin et al. 2005) and d-amino acid oxidase (d-AAO) catalyzed resolution of a methionine racemate (Kohlmann et al. 2009). However, both chemical and electrochemical lead to the inactivation of FADH_2_-dependent enzymes, thus the regeneration rate and TTN are low (Poizat et al. 2010). For enzymatic regeneration, additional NAD(P)H is required to serve as indirect reducing agent and formate dehydrogenase (FDH) is introduced to regenerate NAD(P)H to minimize the cost (Fig. 5d). Hofstetter et al. demonstrated an enzymatic regeneration system for asymmetric cell-free epoxidation with the FADH_2_-dependent monooxygenase StyA and flavin reductase StyB. Their results showed a high TTN value and average reaction rates of StyA in the range of 1800–2800 and 3–4.3 min^−1^, respectively (Hofstetter et al. 2004). In another case, Frese and Sewald developed an enzymatic halogenation system to produce 7-bromo-tryptophan utilizing FADH_2_-dependent L-tryptophan-7-halogenase RebH, flavin reductase PrnF and ADH (Frese and Sewald 2015). For FAD-dependent oxidative deamination, Hou and colleagues constructed a FAD/FADH_2_ regeneration system including FADH_2_-dependent halogenase and FAD-dependent L-amino acid deaminase (L-AAD) to coproduce 7-chloro-tryptophan and indole pyruvic acid (Hou et al. 2023). McGlynn and Fujishima used an O_2_-scavenging cascade which has a similar mechanism to remove O_2_ and produce [4Fe-4S] proteins. Formate dehydrogenase (FDH) was introduced to oxidize formate and was responsible for NADH regeneration. Flavin reductase transferred reducing equivalents and converted FAD to FADH_2_ by consuming NADH. Subsequently, catalase coupled with bifunctional flavin reductase removed O_2_ from the reaction and provided FADH_2_ for [4Fe-4S] protein maturation (Wang et al. 2023).

## Conclusions

CFPS system has advantages to accelerate rapid prototyping and overcoming cellular toxicity. It has been widely applied in the high-throughput production of high-value products (Liu et al. 2019), biosensing (Silverman et al. 2020), integration of orthogonal genetic codes (Chemla et al. 2015), and the assembly of bacteriophages (Shin et al. 2012). With the substantial development of cell free production, low-cost and stable enzymes and cofactor balance and regeneration systems are still the main concern. In this review, we focus on biotechnologically relevant cofactor regeneration systems in CFPS, specifically cofactors found in complex metabolites. The strategies discussed, including ATP regeneration, coenzyme A recycling, and redox cofactor management, represent significant advancements in constructing of cofactor recycling systems and enhancing the production of secondary metabolites. However, there are still challenges for cofactor regeneration systems to be applied in practical production, such as precise dynamic regulation of cofactors, the maintenance of stable cellular redox state, etc. Therefore, the cofactor regeneration technology is expected to be further improved, expanding its application in the field of biocatalysis and biomanufacturing applications. A few examples of success in CFPS systems, where attention to cofactor optimization has led to notable improvements in productivity, would be useful here. Recent advances showed that optimizing the concentration of energy source PEP, and magnesium in CFPS improves the expression of nonribosomal peptide synthetases (Dinglasan et al. 2023). In a developed *Streptomyces* cell-free system, Moore and coworkers investigated most cofactors to identify any nonessential components and optimized the secondary energy source 3-phosphoglyceric acid (3-PGA) and NTP level to have a 52% increase of the protein production (Moore et al. 2021). Many of the pathways described in this review could be integrated into lysates via strategies such as CRISPR/Cas9 so that the enzyme does not need to be added after the fact, sometimes with purification. DNA synthesis and codon optimization also make some of these genes from rare or high GC organisms more accessible. Taken together, new cell free systems, including cofactor balance and lysate composition can be further optimized for utility for secondary metabolite generation which would expand the utility of using these systems for discovery and biosynthetic design prototyping.

## Data Availability

No datasets were generated or analyzed during the current study.
